# Radiation therapy in the adjuvant treatment of hyperkeratotic palmoplantar psoriasis: a case study

**DOI:** 10.1002/jmrs.467

**Published:** 2021-04-01

**Authors:** Bronwyn Shirley, Madeline Andrae, Tegan Le Lay, Michael Collins

**Affiliations:** ^1^ Radiation Therapy Townsville Cancer Centre Townsville Hospital and Health Service Townsville Queensland Australia; ^2^ Royal Marsden Hospital London UK

**Keywords:** Case study, psoriasis, radiation therapy

## Abstract

Psoriasis is an inflammatory autoimmune disease of the skin and nails, causing debilitating pain and having an adverse effect on the patients’ life. Typical treatment regimens involve topical and systemic therapies in combination with phototherapy. However, patients with extensive, chronic disease may encounter treatment resistance, with limited or no success of these therapies. Radiation therapy (RT) has been shown to be an effective treatment for benign skin lesions; however, recommended dose, fractionation and long‐term follow‐up is not well established within the literature making clinical implementation challenging. Furthermore, RT may induce the Koebner Phenomenon, exacerbating the disease. This case study presents a patient with chronic hyperkeratotic palmoplantar psoriasis who was offered RT as a last resort. A total dose of 6Gy was delivered using photons and superficial energies. Significant reduction in extent of disease was seen as a result, with the patient no longer wheelchair‐bound and able to mobilise with minimal discomfort. This case is a single example of RT as a successful treatment for chronic palmoplantar psoriasis; however, a larger sample size and clinical trial is needed to ascertain dose and fractionation for optimal long‐term control. Implementation of such treatments within departments invites clinicians to further develop RT practices and provide much needed relief to a new cohort of patients with non‐malignant conditions.

## Graphical abstract

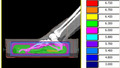

## Introduction

Psoriasis is a chronic skin condition which can be painful, disfiguring and disabling, having an adverse effect on the patients’ quality of life. Psoriasis is considered a serious global problem with a prevalence ranging from 0.09% to 11.4%.^1^ Skin and nail lesions present as localised erythematous patches or plaques of varying sizes which are usually covered in white or silver scales. Further symptoms include itching, fatigue and bleeding if left untreated, potentially leading to psoriatic arthritis, joint deformation and an increased risk of developing cardiovascular and metabolic conditions. Current treatments include concurrent use of topical and systemic therapies, phototherapy (UVB) and Psoralen Photochemotherapy (PUVA). However, these treatments are used solely to manage symptoms, as no cure is available. The use of radiation therapy (RT) in the treatment of benign skin conditions such as psoriasis has been shown to be beneficial; however, long‐term follow‐up is limited within the literature. For patients that have exhausted all alternative options with no or little success, the relatively low risk of receiving low‐dose RT is outweighed by the potential benefits of the treatment. RT can have a carcinogenic effect and even enhance the effect of psoriasis known as the Koebner Phenomenon and should therefore only be used as a last resort in chronic, unmanageable cases.^2^ This case study will highlight the use of RT in a single chronic case of hyperkeratotic palmoplantar psoriasis.

## The Case

**Figure 1:**
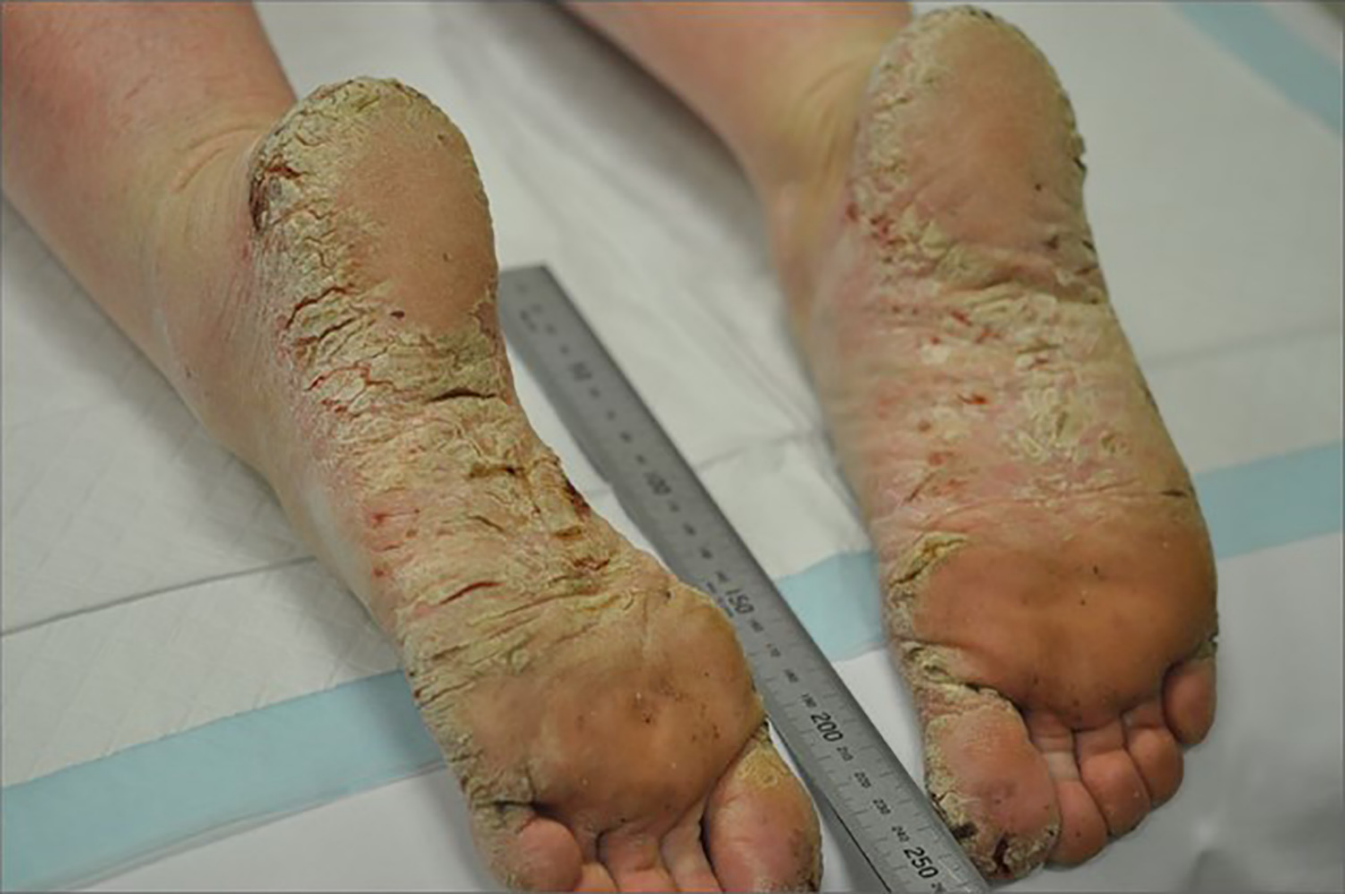
Left and right soles at presentation affected by severe, chronic hyperkeratotic palmoplantar psoriasis prior to receiving radiation therapy.

**Figure 2:**
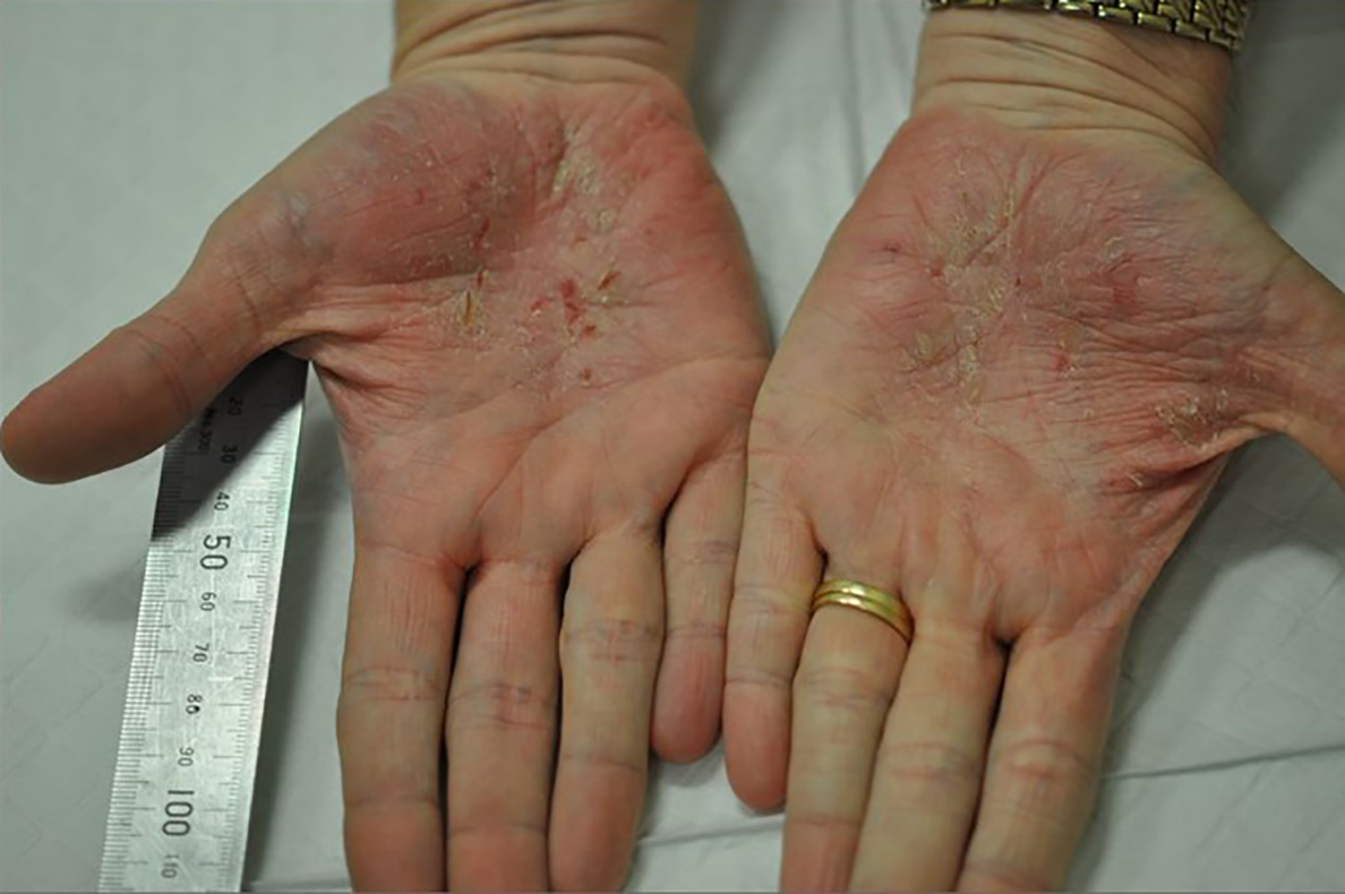
Left and right palms at presentation prior to receiving superficial kilovoltage radiation therapy.

A 39‐year‐old female patient, who has suffered with psoriasis on her scalp from age 5, presented with severe hyperkeratotic palmoplantar psoriasis for over four years. Debilitating pain upon walking had left the patient wheelchair‐bound, unable to work and struggling to care for her six children. Upon assessment, the patient had multiple severe cracking and deep fissures on the soles, heels and toes of both feet, and to a lesser extent on both palms (Figures 1 and 2). Symptomatic control had been attempted through current treatment recommendations including systemic and topical agents to no success and a reduction in liver function. Lesions on the palms measured approximately 4.5cm x 3cm and 0.2cm thick, with lesions on the soles thicker and extending approximately 2cm up the feet and onto the toes. The thickness of the lesions deemed phototherapy and photodynamic chemotherapy (PUVA) unsuitable. RT was offered as a last resort. The patient provided written consent for her case to be presented as a case study. 

### Radiation therapy

**Figure 3:**
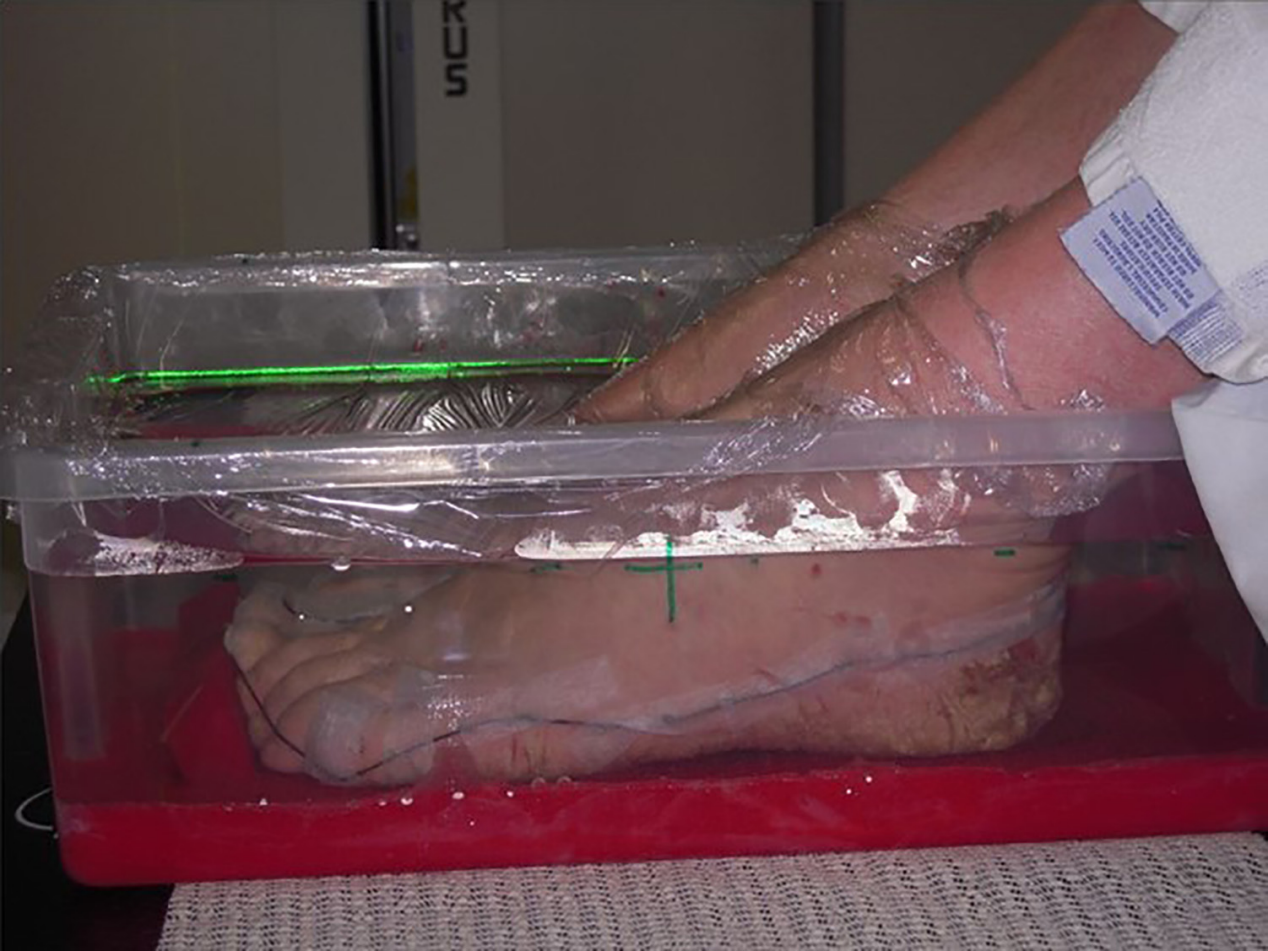
Feet positioned in the custom‐made water bath.

**Figure 4:**
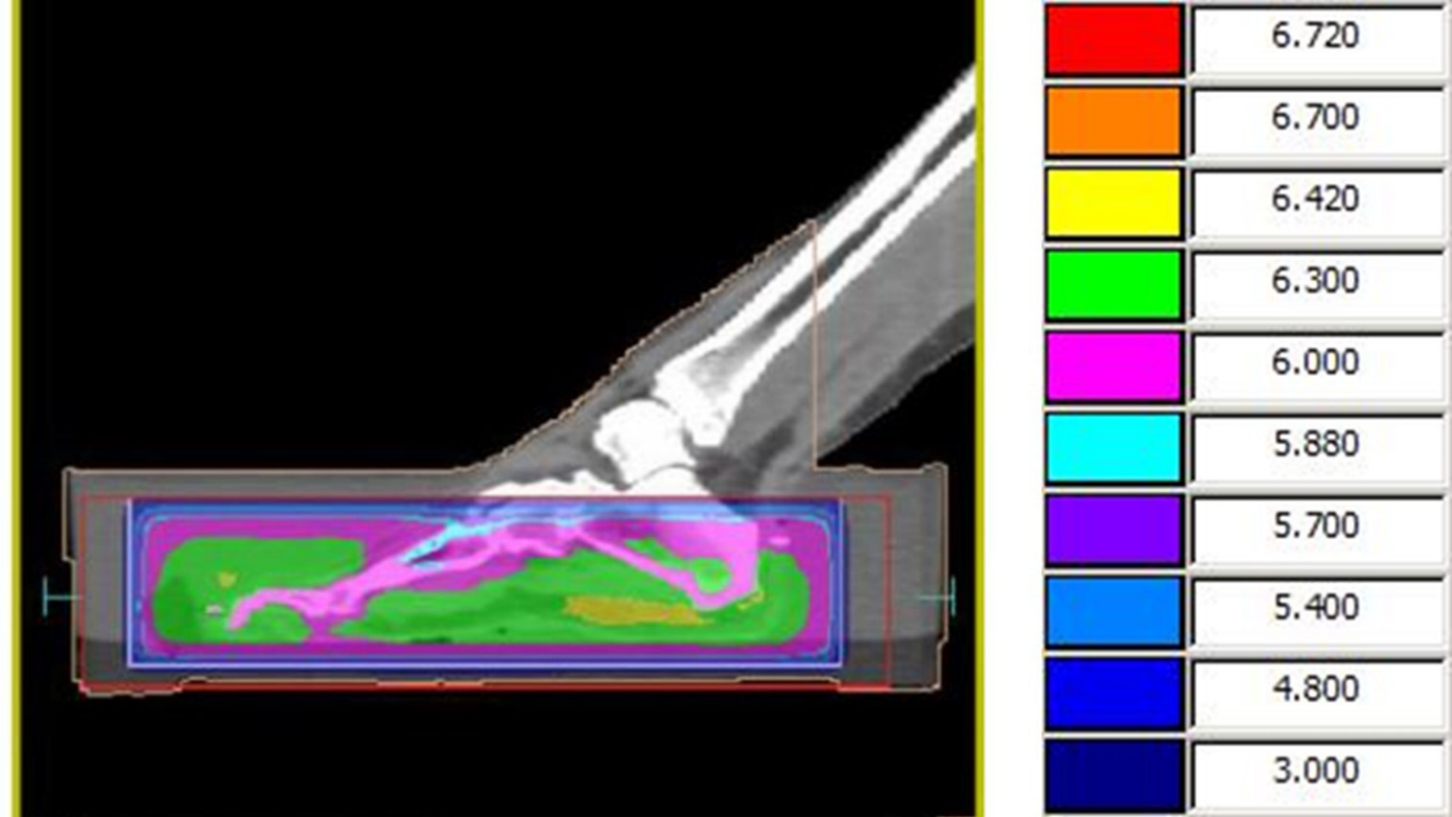
Dosimetry for feet positioned in custom‐made water bath.

Dose and fractionation was agreed upon within a multidisplinary team with evidence reported on by Sumila et al 2008.^3^ A total dose of 6 Gy in 12 fractions was prescribed and delivered to both hands and feet. The patient received two fractions per week at 0.5 Gy per fraction over a 6‐week period. The palms were treated with a direct superficial modality using 70kV energy and a half‐value layer of 1.2mm of aluminium. This energy was chosen due to the equipment limitation with only 70kV and 100kV available to be used. The field encompassed the entire palm and all areas of disease, and no shielding was used. Due to the extensive disease on the feet and the requirement for use of a water bath, 10MV photons opposed lateral technique was used. The feet were positioned on a layer of wax in a custom‐made water bath to overcome the complicated topology and create adequate superficial dose while maintaining homogeniety throughout the treatment volume (Figures 3 and 4). Dose was prescirbed to mid‐plane depth at midline. The Monaco, Elekta treatment planning system was used for this patient due to availability at the treatment centre. Reproducibility was achieved with a tracing of the foot position on the wax with the soles placed flat and distal toes abutting a wax stop. The occurrence of side effects was predicted to be minimal in extent but may have involved the sweat glands and skin. Treatment was based on the individual’s severity at presentation and the small amount of clinical evidence available. Current technology and techniques were utilised, and the treatment was delivered within the normal scope of radiation oncology practice within the department. 

At the time of the fifth fraction, visible improvement was noted on both palms and feet, coinciding with an improvement in patient gait and reduction in pain. By the seventh fraction, some erythema and dryness had developed on the dorsum of the hands; however, the psoriasis had continued to improve. The lesions on the feet appeared to have decreased in thickness, although new cracks were developing. Despite this, the patients’ pain had decreased so significantly that she was able to bear weight and no longer required a wheelchair by the eighth fraction. It was noted by the eleventh fraction that the hands were improving in the treatment area; however, there was progression outside the treatment area on thumbs and fingers.  

### Follow‐up

**Figure 5:**
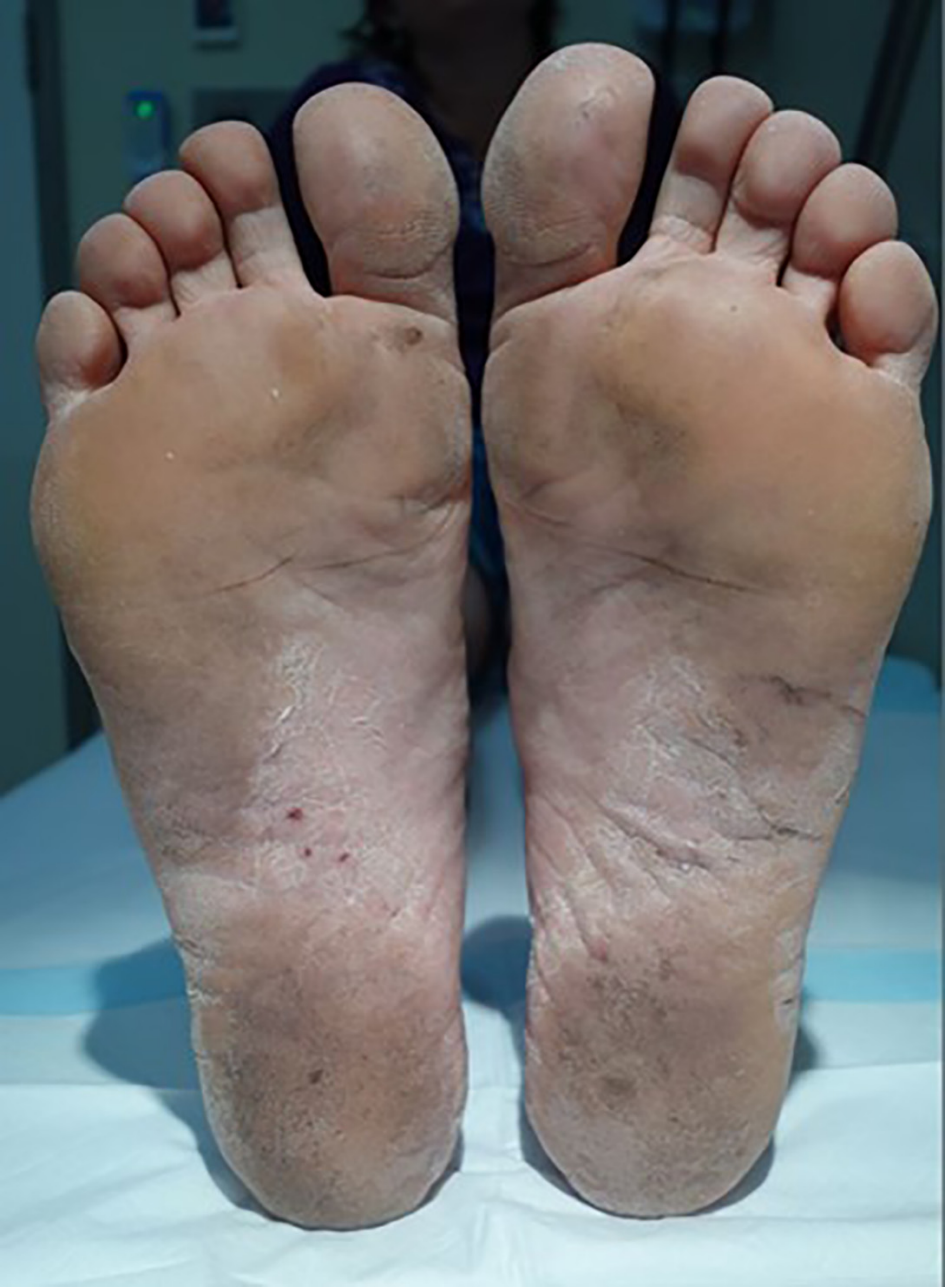
12 months post‐treatment plantar psoriasis.

**Figure 6:**
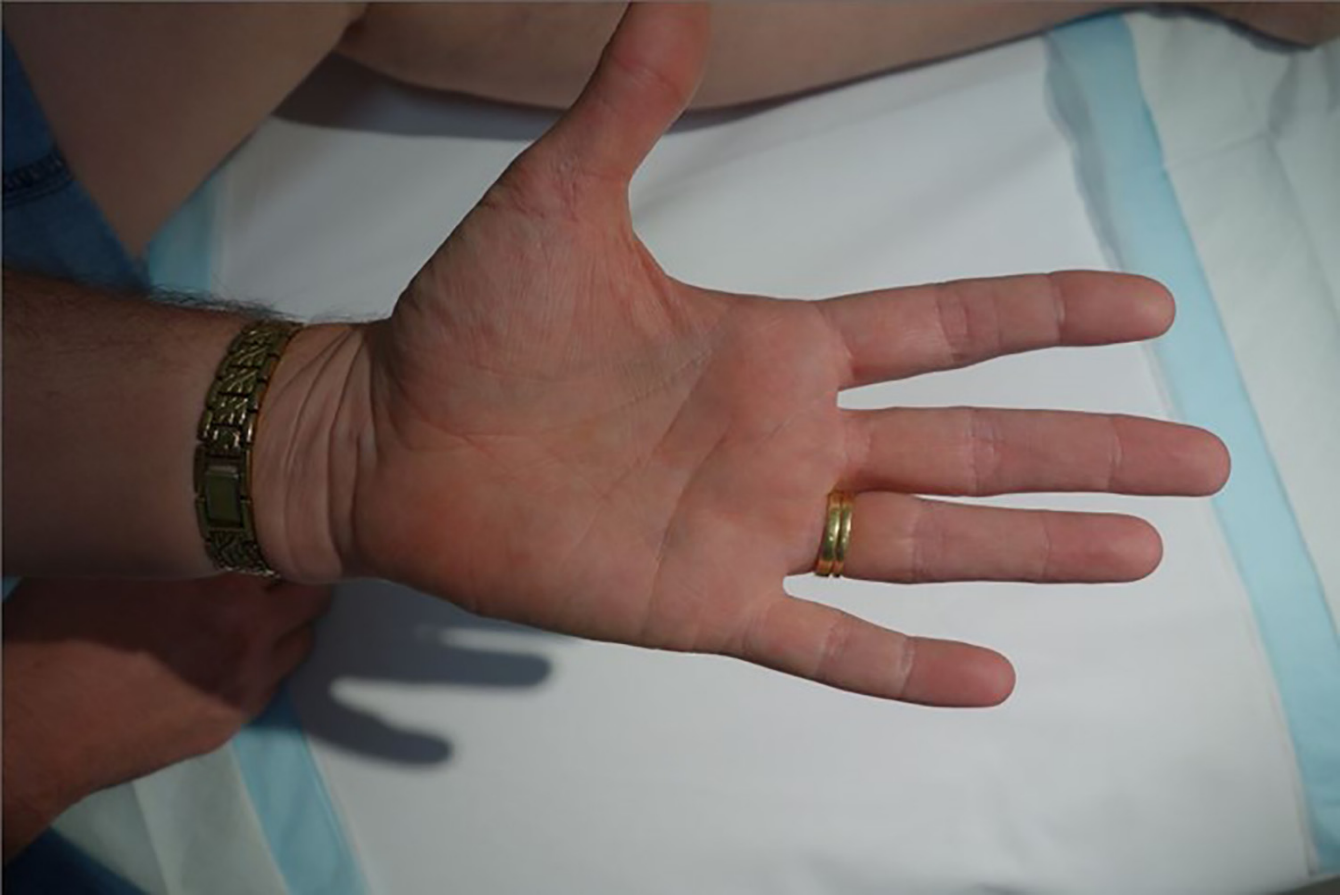
12 months post‐treatment right palmer psoriasis.

**Figure 7:**
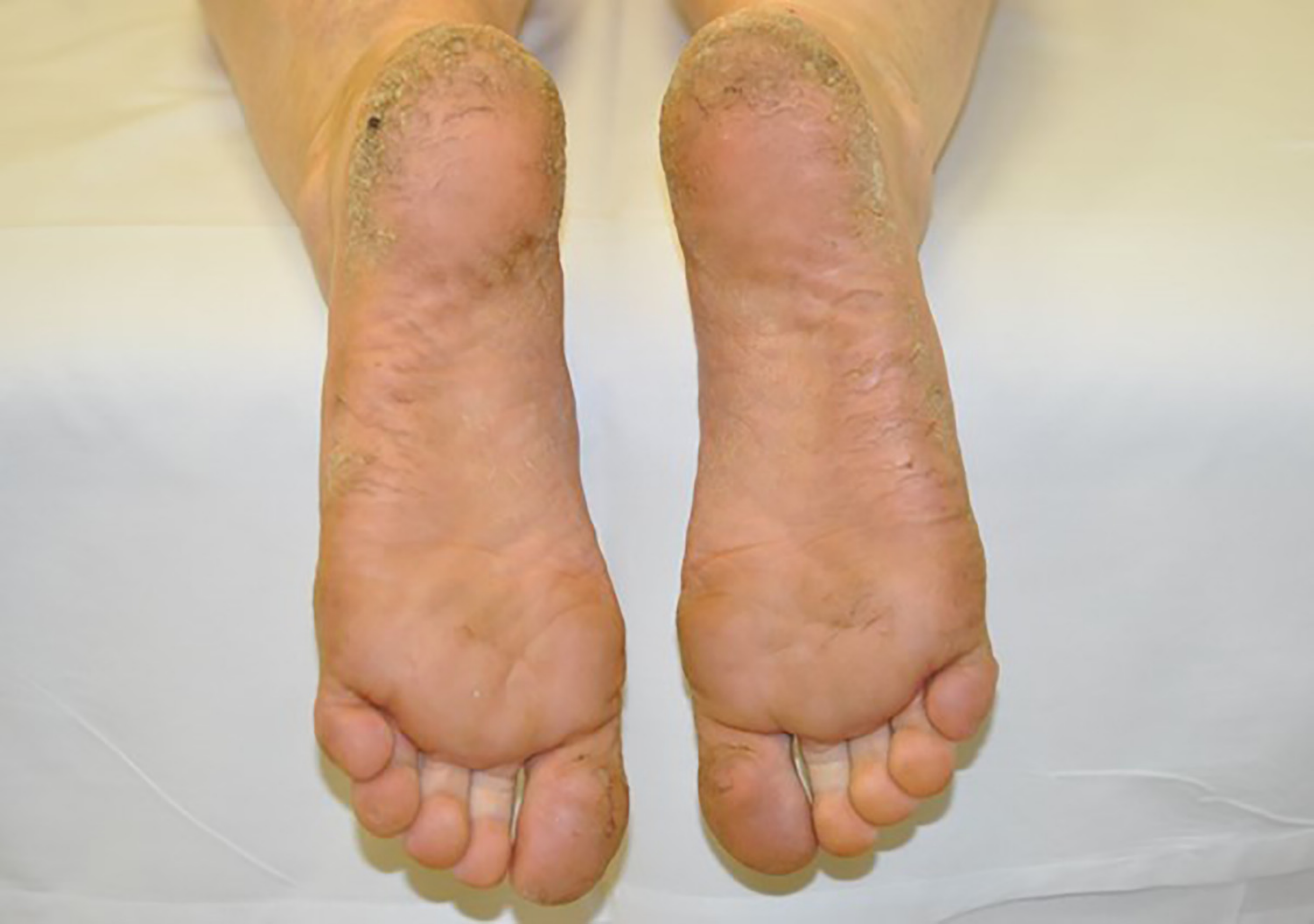
Four years post‐treatment plantar psoriasis.

At the completion of the radiation course, substantial improvement within the treatment areas was seen, and the patient could walk with minimal pain and was satisfied with the results. Post‐RT, the patient reported continuing good results, with her psoriasis able to be managed with the conventional systemic and topical therapies that were previously ineffective and intolerable. Four months post‐treatment, there was no visible disease on the palms; however, some cracking was present outside of the field. There was some cracking on the heels yet no evidence of skin breakdown or pain, and the patient could wear shoes with no discomfort. Eight months post‐treatment, there was no detectable hyperkeratosis on either hand and she was still able to independently mobilise with little discomfort. Some reports of peripheral keratosis were made, however, were attributed to the cooler weather. Twelve months post‐treatment, the palmoplantar psoriasis remained under control (Figures 5 and 6). At sixteen months post‐treatment, the patient experienced a flare in the disease to the feet which was followed up and successfully treated by a dermatologist using medication that again, before treatment, was ineffective. Upon resolution, she continued to mobilise independently with no pain, paraesthesia or functionality issues in either hands and feet. She is now being followed up annually with the radiation oncologist. At two years post‐treatment, she had experienced some plantar flare ups which was further controlled by her dermatologist and altering her medication. Palmer psoriasis remained under control. At two and half years post‐treatment follow‐up, plantar psoriasis was more uncomfortable, and this may have been due to the decrease in immunosuppressive drug and trial of an alternative drug; however, this was controlled within 12 months at the three and a half year follow‐up. Most recently, at four years post‐treatment, the patient reported that cracked skin on her feet was worse but raw tissue was minimal with some hypokeratosis, and no pain. No significant change was evident on her palms. Even though this patient still requires immunosuppressive therapy, her plantar psoriasis is now moderate and she is able to carry out daily activities, and walk without using a wheelchair (Figure 7).

## Discussion

By altering the dose and fractionation of RT to much lower than that typically used for malignant cancers (<1 Gy per fraction), anti‐inflammatory effects occur and can be utilised to treat a range of inflammatory, benign conditions.^4^ However, the relationship between low‐dose ionising radiation and the cellular and molecular mechanisms involved in inflammatory skin conditions is extremely complex and not well understood. Fractionated doses of less than 1 Gy to a total dose of 6 Gy have been shown to inhibit well established inflammatory processes such as those seen in chronic psoriasis, hence providing a therapeutic effect.^5^ In vivo studies completed on rabbit and rat models support this low dose and fractionation showing a reduction in inflammation and associated symptoms when exposed to 0.5–2 Gy per treatment to a total dose of most commonly 5 Gy.^4^ Despite this, dose regimens recommended specifically for chronic psoriasis are limited by a lack of rigorous scientific evidence. Although the available literature discusses similar dose and fractionations, it fails to recommend a specific RT regimen for ultimate long‐term control.

In this case study, dose and fractionation was determined in conjunction with multiple departmental multidisciplanary team meetings and evidence reported on by Sumila et al 2008.^3^ Sumila et al 2008 concluded that excellent results can be achieved in the treatment of palmoplantar eczema or psoriasis when treating 0.5 Gy twice a week with a total dose of 4–5 Gy. They also highlight that all previously recorded randomised control trials fail to satisfy current standards due to low cohort numbers and short follow‐up of only 1.3–6 months.^3^ Their results show no significant differences between patients who received either a fractionated dose of 1 Gy to a total dose of 7–13 Gy, and fractionated doses of 0.5 Gy to a total dose of 2.5–8 Gy. Both regimens were seen to have substantial rates of improvement, including some patients reporting total remission.

Patients treated with RT are also at risk of exacerbating the symptoms due to the Koebner Phenomenon.^2^ It is not well understood; however, it is proposed that the skin cell damage caused by the radiation treatment could cause a Psoriatic flare up in the treatment area, resulting in the development of new lesions. Due to this, it is crucial that patients have attempted all conventional and alternative treatment options prior to considering RT as a last resort for unmanageable, severe psoriasis. In this case study, the potential benefits outweighed the risk of any minor flare ups that could have been attributed to the Koebner Phenomenon.

The efficiency of open‐field radiation as a treatment for benign palmoplantar skin conditions can be limited by the complicated topology of the feet and hands. However, using custom‐made applicators to deliver high dose rate brachytherapy may be a way to overcome this. Computer‐optimised HDR brachytherapy has the capability to deliver a highly conformal, homogenous dose with a steep fall‐off gradient to the superficial dermis only. Advantageous over open field, the underlying healthy tissue and bone would be spared, decreasing the risk of radiation‐induced side effects and increasing the specificity of treatment.^6^ The utilisation of this type of treatment for psoriasis is extremely limited in the literature and discusses psoriatic nail beds and palmoplantar pustular dermatitis exclusively. Furthermore, each case differs significantly in fractionation and total dose, with a maximum reported total dose of 21.6 Gy.^7^ The lack of clinical evidence and guidelines makes implementation of the treatment within new departments challenging. Further challenges arise with the design and manufacture of the applicators, accessibility to equipment, dosimetry characteristics including optimisation with non‐standard applicators, immobilisation and the availability of trained staff and facilities. For these reasons, HDR brachytherapy was considered infeasible as a treatment option within our department.

## Conclusion

More research is needed in determining optimal dose/fractionation and benefits of RT in treatment of inflammatory, benign skin diseases such as psoriasis. Given the small cohort of patients with severe and debilitating disease warranting a more extreme treatment such as RT, it is difficult to ascertain a large sample for clinical study. Despite this, our case study identifies a single success in the treatment of resistant hyperkeratotic palmoplantar psoriasis utilising currently recommended dose regimes. Increased quality of life has shown the success of this treatment for this particular patient. The once debilitating disease is now managed with standard therapy; however, the low prescribed RT dose still allows for future RT if disease progression occurs. Future work will aim to contribute to a larger national/international database, looking to develop a more comprehensive radiation therapy treatment regime for benign skin diseases.

## Conflict of Interest

The authors declare no conflict of interest.

## References

[1] WHO . Global Report on Psoriasis. World Heal Organ are available WHO website or can be Purch from WHO Press World Heal Organ 2016;48.

[2] Ben‐Yosef R, Soyfer V, Vexler A. Radiation therapy in cancer patients with psoriasis. The fractionated daily dose and the Koebner phenomenon. Radiother Oncol 2005; 74: 21–3. 15683664 10.1016/j.radonc.2004.08.019

[3] Sumila M, Notter M, Itin P, Bodis S, Gruber G. Long‐term results of radiotherapy in patients with chronic palmo‐plantar eczema or psoriasis. Strahlentherapie und Onkol 2008; 184: 218–23. 10.1007/s00066-008-1788-4 18398587

[4] The Royal College of Radiologists . A review of the use of radiotherapy in the UK for the treatment of benign clinical conditions and benign tumours. Clin Oncol 2015; 1: 115.

[5] Frey B, Hehlgans S, Rödel F, Gaipl US. Modulation of inflammation by low and high doses of ionizing radiation: implications for benign and malign diseases Modulating Responses. Cancer Lett 2018; 368: 230–7. 10.1016/j.canlet.2015.04.010 25888451

[6] Timerman D, Devlin PM, Nambudiri VE, et al. Novel application of high‐dose rate brachytherapy for severe, recalcitrant palmoplantar pustulosis. Clin Exp Dermatol 2016; 41: 498–501. 26848819 10.1111/ced.12803 PMC5317041

[7] Buzurovic IM, O’Farrell DA, Bhagwat MS, et al. Custom‐made micro applicators for high‐dose‐rate brachytherapy treatment of chronic psoriasis. J Contemp Brachytherapy 2017; 9: 263–9. 28725251 10.5114/jcb.2017.68304 PMC5509984

